# BAP31 represses endoplasmic reticulum stress-mediated apoptosis and alleviates neurodegeneration in Parkinson’s disease

**DOI:** 10.1038/s41419-025-07907-3

**Published:** 2025-09-29

**Authors:** Yan Qin, Ying Chen, Minghao Chi, Junzhen Jia, Li Wang, Yan Zhao, Zhecheng Wang, Junjun Zhou, Jihong Yao

**Affiliations:** Department of Pharmacology, Dalian Medical University, Dalian, China

**Keywords:** Parkinson's disease, Parkinson's disease, Neural ageing

## Abstract

Excessive endoplasmic reticulum (ER) stress and neuronal apoptosis contribute to neurodegeneration in Parkinson’s disease (PD). However, the molecular mechanisms underlying these perturbations and how they are directly regulated remain unclear. B cell receptor-associated protein 31 (BAP31), which is highly expressed in the ER, has been shown to participate mainly in regulating ER stress and apoptosis. Here, our results showed that BAP31 expression was dramatically decreased in PD. Notably, overexpression of BAP31 exerted neuroprotective effects by inhibiting ER stress and apoptosis in vitro and in vivo, whereas BAP31 siRNA strongly abolished these effects. Interestingly, 4-phenylbutyric acid (4-PBA), the ER stress inhibitor, reversed the detrimental effect of BAP31 knockdown in vitro. Mutations in PTEN-induced putative kinase 1 (PINK1) are known to cause autosomal recessive early-onset PD. PINK1 has been implicated in protein phosphorylation pathways that are associated with ER stress and apoptosis. Bioinformatics analysis and our results demonstrated that PINK1 interacts with BAP31 and phosphorylates it at the Ser 142 residue. Furthermore, the protective effects of PINK1 overexpression against ER stress-mediated apoptosis were abolished by BAP31 interference or BAP31-S142A and strengthened by BAP31-S142E. Overall, the present study suggests that BAP31 overexpression exerts neuroprotective effects by inhibiting ER stress-induced apoptosis. Regulation of the PINK1/BAP31 pathway may be a beneficial strategy for PD.

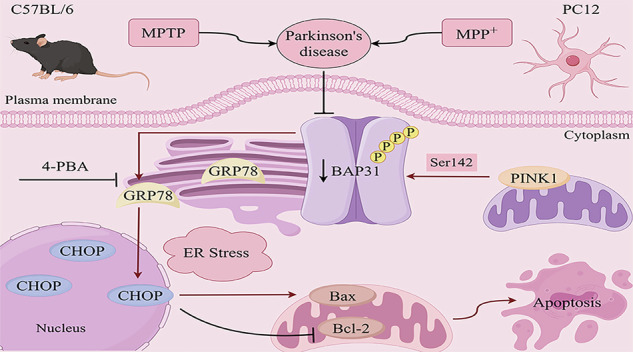

## Introduction

Parkinson’s disease (PD), which is one of the fastest-growing neurodegenerative diseases among elderly people in the world, is characterized by the progressive loss of dopaminergic (DA) neurons in the substantia nigra (SN) [1, 2]. Loss of DA neurons results in declines in motor function, including bradykinesia, resting tremor, rigidity, postural and gait instability, as well as cognitive deficits and autonomic dysfunction [3]. Despite ongoing research, the mechanisms underlying PD remain poorly understood. Recent studies highlighted the critical roles of endoplasmic reticulum (ER) stress and neuronal apoptosis in PD pathogenesis [4, 5].

ER stress is generally activated in response to various stressful conditions, including alterations of calcium homeostasis and redox status, hypoxia, low glucose levels and abnormally high cholesterol levels, as well as genetic factors such as specific gene mutations; collectively, these factors lead to the accumulation of unfolded/misfolded proteins in the ER lumen [6–8]. Moreover, prolonged ER stress may disrupt the protective mechanism of the unfolded protein response (UPR) and activate an apoptotic program [9, 10]. Recent studies have demonstrated that ER stress also disrupts functional interactions between the ER and mitochondria at specialized membrane contact sites (mitochondrial-associated ER membranes, MAMs). Perturbations in the ER-mitochondria association have been implicated in PD pathogenesis [11, 12]. Numerous proteins associated with PD, including alpha-synuclein (α-syn), Parkin RBR E3 ubiquitin-protein ligase (Parkin), PTEN-induced putative kinase 1 (PINK1), and DJ-1, are localized in MAMs and alter ER-mitochondria association [13–16]. Importantly, excessive induction of ER stress and apoptosis contributes significantly to PD-associated neuronal loss. In contrast, inhibition of ER stress and apoptosis promotes tyrosine hydroxylase (TH)-positive cell survival and alleviates DA neuronal injury in the SN, ultimately ameliorating the characteristics of PD in cellular and animal models [17–20]. Therefore, ER stress and apoptosis play pivotal roles in the neurodegeneration of PD. Investigating critical modulators of ER stress and apoptosis is an essential strategy for PD treatment.

B cell receptor-associated protein 31 (BAP31) is an ER membrane protein that is predominantly localized to the ER and highly expressed in neurons. In recent years, attention has focused on the regulatory effects of BAP31 on various biological functions, including ER stress and apoptosis [21, 22]. The evidence has indicated that full-length BAP31 can directly inhibit the apoptotic pathway initiated by caspase-8 [23]. Moreover, BAP31 has been shown to suppress ER stress by interacting with the apoptosis protein cell death-inducing p53 target 1 (CDIP1) [24]. Upregulation of BAP31 attenuates ER stress and mitochondrial dysfunction-mediated apoptosis in lipopolysaccharide (LPS)-induced alveolar epithelial type II (ATII) cells [25]. Furthermore, previous work from our laboratory has demonstrated that deacetylation of BAP31 by sirtuin 2 attenuates ER stress-induced apoptosis in chronic alcoholic liver injury [26]. Notably, BAP31 may act as a critical neuroprotective regulator in the central nervous system. BAP31 deficiency exacerbates LPS-induced microglial activation and neuronal death via IL-1 receptor-associated kinase (IRAK1) [27], and promotes amyloid-β plaque formation in Alzheimer’s disease (AD) mouse model through destabilization of reticulon 3 [28]. Mutations in BAP31 cause an X-linked syndrome, which is characterized by progressive neurological deterioration, such as motor and intellectual disabilities, and dystonia [29]. However, the role of BAP31 in PD remains poorly understood.

In our study, we report for the first time that BAP31 expression is dramatically decreased in PD both in vivo and in vitro. The overexpression of BAP31 remarkably inhibits ER stress-induced apoptosis. Moreover, BAP31 is phosphorylated by PINK1 (a key pathogenic gene in autosomal recessive early-onset Parkinson’s syndrome) at the serine 142 residue, which alleviates PD by inhibiting ER stress-mediated apoptosis. These findings demonstrate a novel neuroprotective effect of BAP31 and suggest that focusing on the PINK1/BAP31 pathway may provide an innovative strategy for PD treatment.

## Results

### Decreased expression of BAP31 in PD

**Fig. 1:**
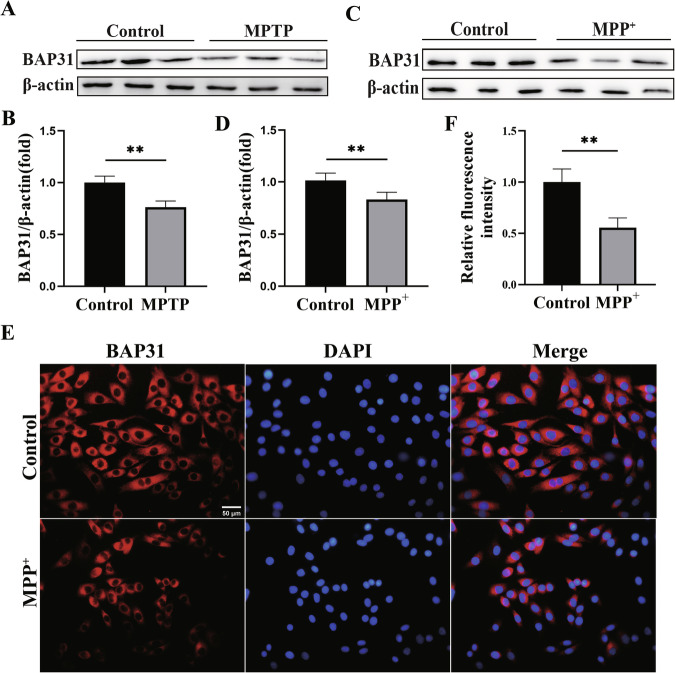
Decreased expression of BAP31 in PD. **A**, **B** Expression of BAP31 protein in MPTP-induced PD mice (*n* = 3). **C**, **D** Expression of BAP31 protein in MPP^+^-induced PC12 cells (*n* = 3). **E**, **F** Subcellular localization and immunofluorescence quantification of BAP31 (Scale bar = 50 μm). **P < 0.01.

To investigate the role of BAP31 in PD, we first measured the expression of BAP31 both in vitro and in vivo by Western blotting. As shown in Fig. 1A, B, the expression of BAP31 was significantly decreased in the 1-methyl-4-phenyl-1,2,3,6-tetrahydropyridine (MPTP)-induced PD mice. Similarly, BAP31 expression was notably decreased in 1-methyl-4-phenylpyridinium (MPP^+^)-induced PC12 cells (Fig. 1C, D). Furthermore, we confirmed the subcellular localization of BAP31 using immunofluorescence. The results revealed that BAP31 was located primarily in the cytoplasm, and its fluorescence was markedly reduced in MPP^+^-treated PC12 cells (Fig. 1E, F). Collectively, our results indicate that BAP31 expression is obviously decreased in PD.

### Overexpression of BAP31 significantly alleviated PD

**Fig. 2:**
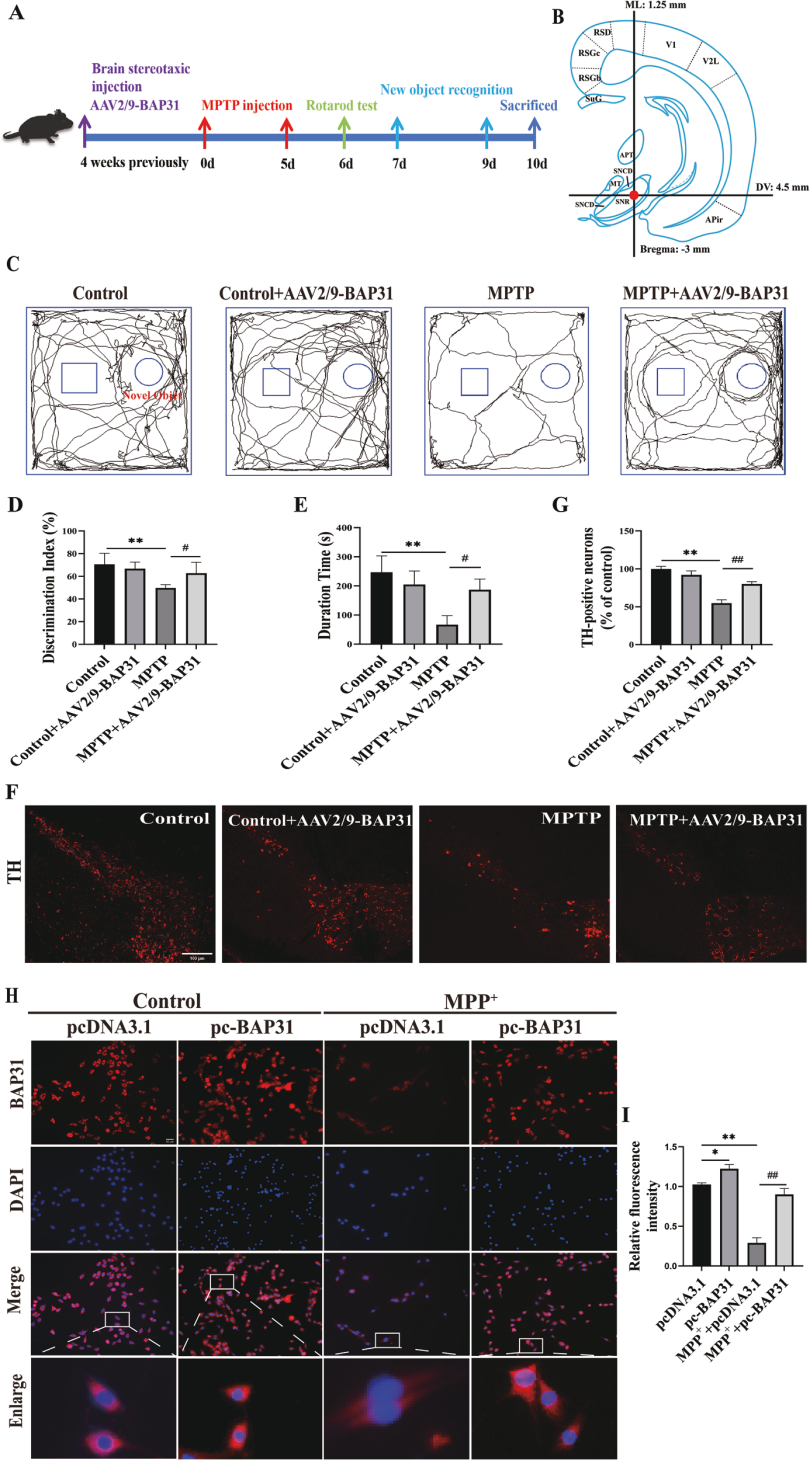
Overexpression of BAP31 significantly alleviated PD. **A**, **B** Experimental flowchart and stereotactic injection coordinates of SN. **C** Novel object recognition exploratory trajectory (*n* = 10). **D** Novel object recognition index (*n* = 10). **E** Rotarod test (*n* = 10). **F**, **G** The number of TH-positive neurons in the SN of PD mice (Scale bar = 100 μm). **H**, **I** BAP31 subcellular localization and expression in MPP^+^ treated PC12 cells (Scale bar = 50 μm). *P < 0.05, **P < 0.01, ^#^*P* < 0.05, ^##^*P* < 0.01.

Next, to further validate the protective effect of BAP31 on PD, we generated BAP31-overexpressing mice using AAV2/9-BAP31 (Fig. 2A, B). In the behavioral tests, overexpression of BAP31 significantly improved the memory ability of PD mice (Fig. 2C, D) and reversed the decreased latency to fall in the rotarod test (Fig. 2E). Furthermore, we used immunofluorescence staining to examine the expression of TH, which is the rate-limiting enzyme in the synthesis of dopamine in dopaminergic neurons. Notably, the loss of TH expression in MPTP-treated PD mice was obviously reversed in BAP31-overexpressing mice (Fig. 2F, G). Additionally, the overexpression of BAP31 apparently enhanced fluorescence in MPP^+^-treated PC12 cells (Fig. 2H, I). Altogether, these findings reveal that the overexpression of BAP31 dramatically alleviates PD injury and exerts neuroprotective effects.

### BAP31 is indispensable for regulating ER stress-mediated apoptosis in PD

**Fig. 3:**
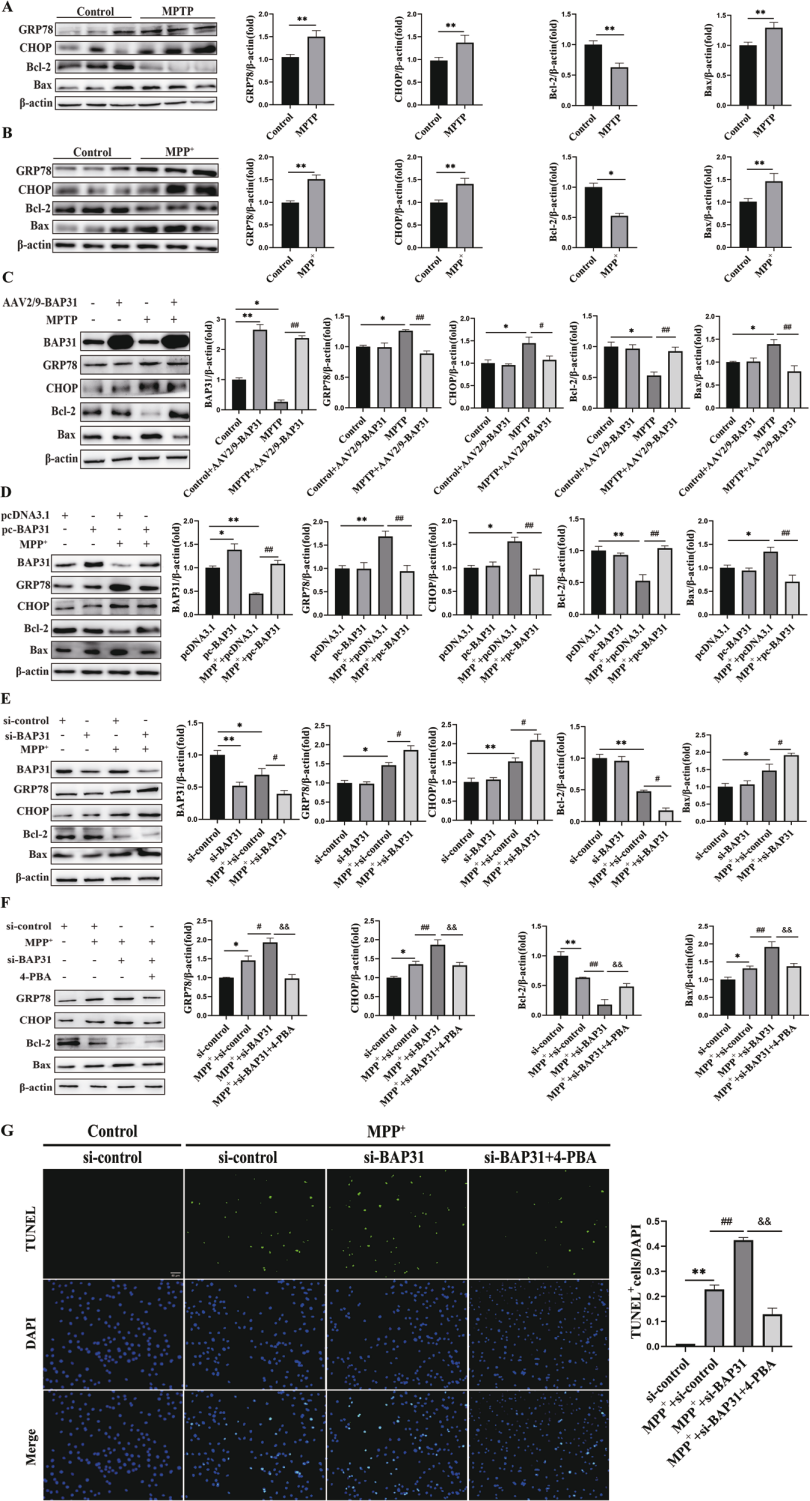
BAP31 is required for resistance to ER stress-mediated apoptosis in PD. **A** ER stress and apoptotic proteins expressions in MPTP-induced PD mice (*n* = 3). **B** ER stress and apoptotic proteins expressions in MPP^+^-induced PC12 cells (*n* = 3). **C** Overexpression of BAP31 inhibits ER stress and apoptosis in PD mice (*n* = 3). **D** Overexpression of BAP31 suppresses ER stress and apoptosis in PC12 cells (*n* = 3). **E** Interference with BAP31 promotes ER stress and apoptosis in PC12 cells (*n* = 3). **F** The ER stress inhibitor 4-PBA inhibits ER stress and apoptosis (*n* = 3). **G** TUNEL staining (Scale bar = 50 μm). *P < 0.05, **P < 0.01, ^#^*P* < 0.05, ^##^*P* < 0.01, ^&&^*P* < 0.01.

In order to investigate whether BAP31 mediates the effects of ER stress and apoptosis on PD, we first measured the protein expression of markers of ER stress and apoptosis, including glucose-regulated protein 78 (GRP78), C/EBP-homologous protein (CHOP), Bcl-2-associated X protein (Bax) and B-cell lymphoma-2 (Bcl-2) in vivo and in vitro via Western blotting. Compared to the control group, the expressions of GRP78, CHOP, and Bax were significantly increased, whereas Bcl-2 expression was markedly decreased in the MPTP/MPP^+^-induced PD model (Fig. 3A, B). More strikingly, BAP31-overexpressing mice presented markedly reduced GRP78, CHOP, and Bax protein expression and increased Bcl-2 protein expression after MPTP treatment (Fig. 3C). Similarly, the results of BAP31 overexpression in PC12 cells were consistent with those described above (Fig. 3D). Conversely, ER stress and apoptosis during MPP^+^ treatment were exacerbated by BAP31 knockdown in PC12 cells (Fig. 3E). Next, to further evaluate the effect of BAP31 on PD through ER stress-induced apoptosis, we used 4-phenylbutyric acid (4-PBA) as an ER stress inhibitor [30]. We found that the expression of the ER stress-mediated apoptosis markers GRP78, CHOP, and Bax was significantly reduced, whereas the expression of Bcl-2 was clearly elevated by 4-PBA in the BAP31 knockdown group (Fig. 3F). Consistently, the TUNEL assay results demonstrated that 4-PBA attenuated the amelioration of PD injury caused by BAP31 knockdown (Fig. 3G). In summary, our data confirm that BAP31 plays a vital role in ER stress-mediated apoptosis in PD, and that suppressing ER stress-mediated apoptosis could be a promising strategy to prevent PD.

### BAP31 is phosphorylated by PINK1 at Ser142

**Fig. 4:**
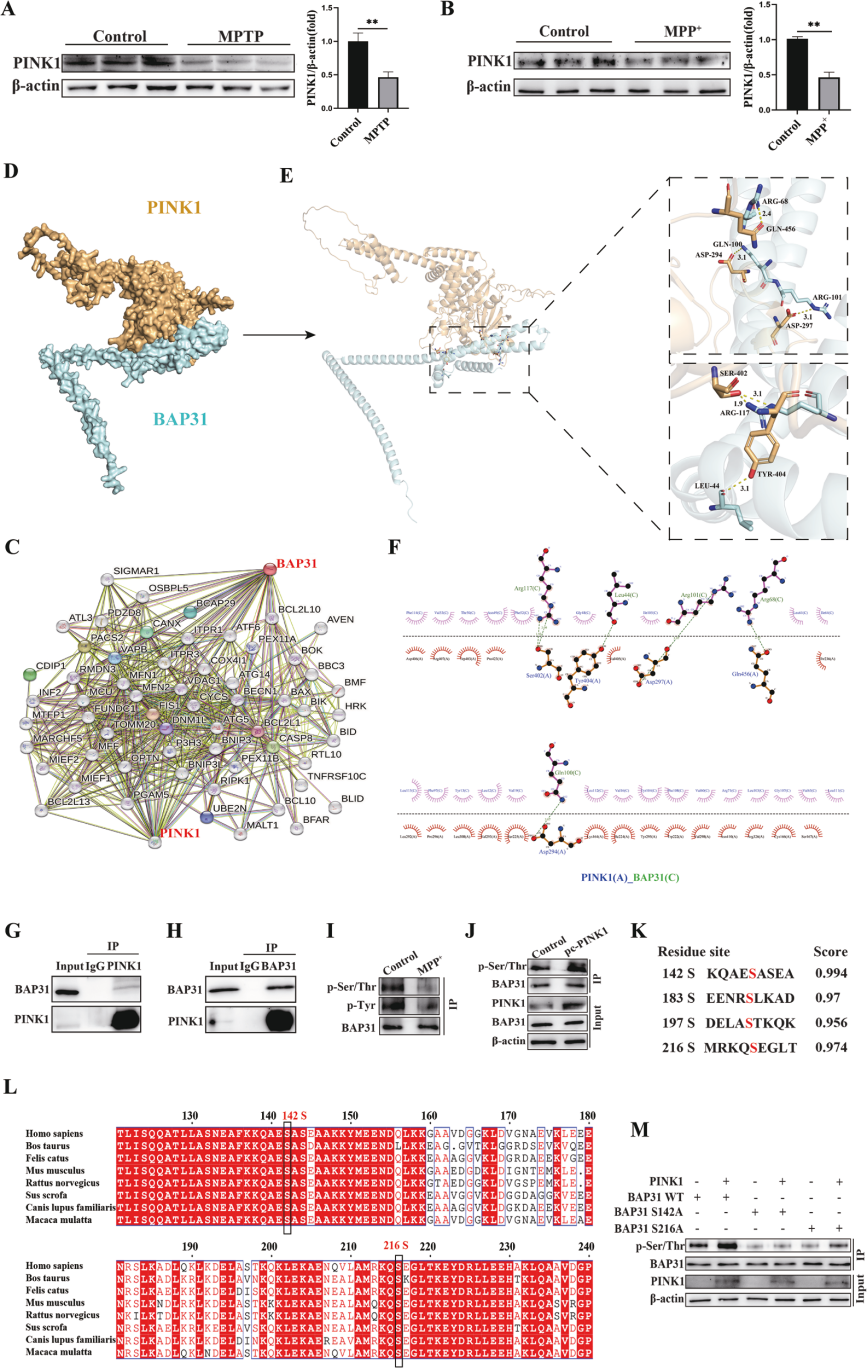
BAP31 is phosphorylated by PINK1 at Ser 142. **A**, **B** The protein expression of PINK1 in vivo and in vitro (*n* = 3). **C** String database. **D–F** Molecular docking. **G**, **H** Co-immunoprecipitation validates PINK1 interaction with BAP31 (*n* = 3). **I** Immunoprecipitation verifies the phosphorylation of BAP31 (*n* = 3). **J** Overexpression of PINK1 impacts BAP31 phosphorylation in PC12 cells (*n* = 3). **K** NetPhos 3.1 database analysis. **L** Comparative analysis of amino acid sequences around the site among different species. **M** Immunoprecipitation analysis in PC12 cells after co-transfection of BAP31 (WT, Ser142A and Ser216A) and PINK1 (*n* = 3). **P < 0.01.

Mutations in PINK1 are known to cause autosomal recessive early-onset PD. The PINK1 gene encodes a mitochondrial serine/threonine protein kinase that interacts with substrate proteins and phosphorylates them [31, 32]. We first confirmed by Western blotting that PINK1 protein expression was notably decreased in PD (Fig. 4A, B). More importantly, the vital role of BAP31 in ER stress-mediated apoptosis in PD prompted us to examine the correlation between PINK1 and BAP31. The results of the STRING database (http://stringdb.org/) prediction indicated that BAP31 potentially interacts with PINK1 (Fig. 4C), and molecular docking was used to further validate this interaction (Fig. 4D–F). The interaction of PINK1 with BAP31 was subsequently confirmed by co-immunoprecipitation (Co-IP) assay (Fig. 4G, H). Next, we explored whether PINK1 influences the phosphorylation of BAP31. To address this question, we determined the level of BAP31 phosphorylation via immunoprecipitation (IP). As shown in Fig. 4I, J, the phosphorylation of BAP31 was obviously decreased in MPP^+^-induced PC12 cells, whereas the overexpression of PINK1 notably increased the phosphorylation of BAP31. To further determine the specific site of PINK1-regulated BAP31 phosphorylation, the amino acid sequence of BAP31 was analyzed by the NetPhos 3.1 (https://services.healthtech.dtu.dk/services/NetPhos3.1/). Sequence analysis revealed two potential phosphorylation sites of BAP31, Ser142 and Ser216 (Fig. 4K), which are particularly conserved across species (Fig. 4L). Next, we created BAP31 mutants by mutating serine (S) to alanine (A) at both Ser142 and Ser216. Interestingly, the phosphorylation of BAP31 was reduced in both the S142A and S216A mutants. More significantly, the overexpression of PINK1 obviously increased the phosphorylation of BAP31 at the S216A mutation site but not at the S142A mutation site (Fig. 4M), indicating that Ser142 is an essential site for PINK1-dependent BAP31 phosphorylation. Overall, these findings suggest that PINK1 interacts with BAP31 and phosphorylates BAP31 at Ser142.

### PINK1 inhibits ER stress-mediated apoptosis in PD by phosphorylating BAP31 at Ser142

**Fig. 5:**
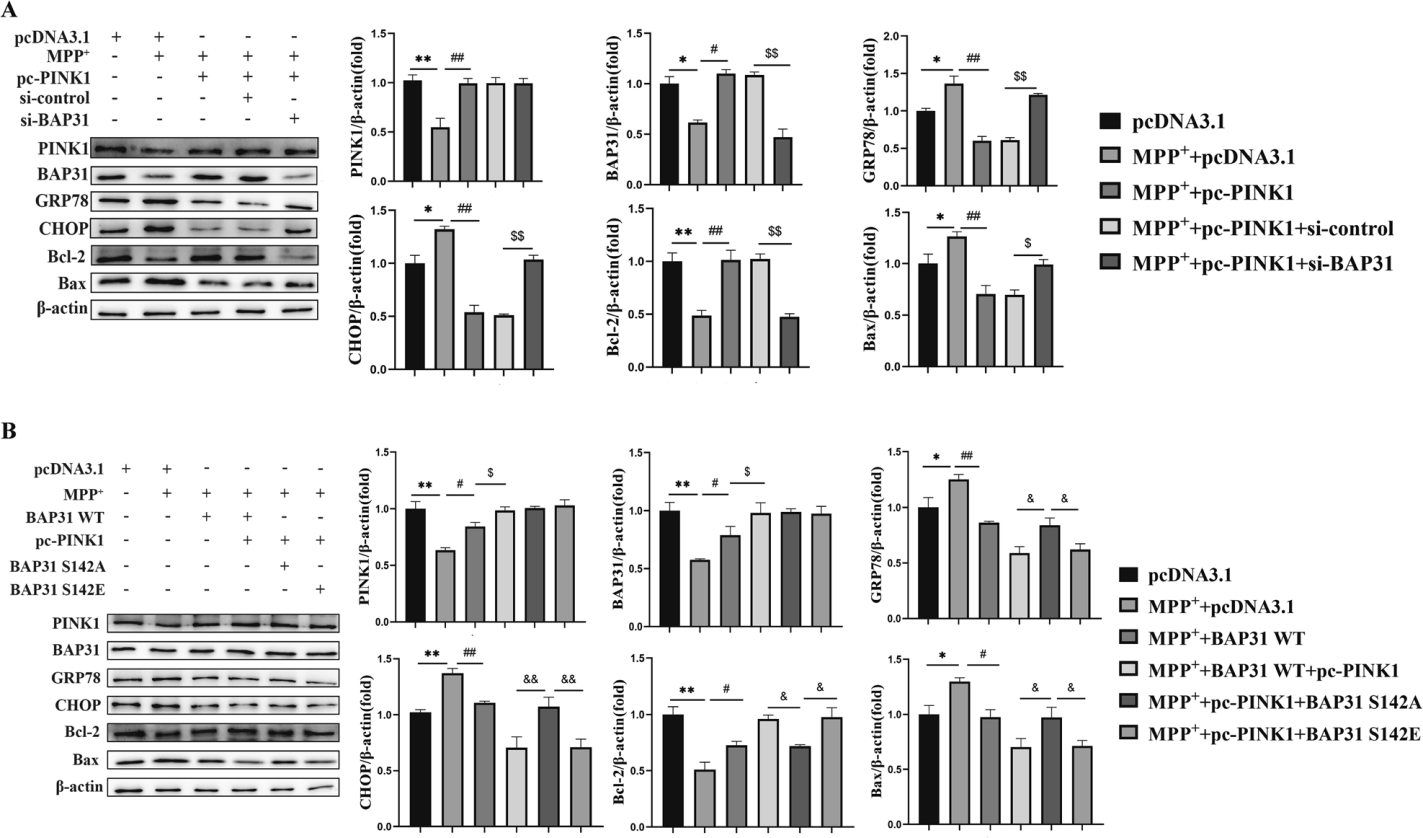
PINK1 inhibits ER stress-mediated apoptosis in PD through BAP31 phosphorylation at Ser142. **A** ER stress and apoptotic protein expressions by co-transfecting with PINK1 overexpression and BAP31 interference in MPP^+^-induced PC12 cells (*n* = 3). **B** ER stress and apoptotic protein expressions by co-transfecting with PINK1 and BAP31 (WT, Ser142A and Ser142E) in MPP^+^-induced PC12 cells (*n* = 3). *P < 0.05, **P < 0.01, ^#^*P* < 0.05, ^##^*P* < 0.01, ^$^*P*^<^0.05, ^$$^*P* < 0.01, ^&^*P* < 0.05, ^&&^*P* < 0.01.

Considering that BAP31 is indispensable for regulating ER stress-mediated apoptosis in PD and can be phosphorylated by PINK1 at Ser142, we first overexpressed PINK1 and knocked down BAP31 in PC12 cells to assess the effect on ER stress- and apoptosis-related protein expression in PD. Notably, the overexpression of PINK1 dramatically reduced the expression of GRP78, CHOP, and Bax while promoting the expression of Bcl-2, whereas knocking down BAP31 reversed the protective effects of PINK1 overexpression against ER stress and apoptosis in PD (Fig. 5A). Next, we determined whether PINK1 regulates ER stress-mediated apoptosis by phosphorylating BAP31 at Ser142. PC12 cells were co-transfected with PINK1 overexpression and BAP31-WT (wild-type), BAP31-S142A (phosphorylation-dead), or BAP31-S142E (phosphorylation-mimic) mutants in PD. Of interest, compared to BAP31-S142A, BAP31-WT and BAP31-S142E enhanced the protective effects of PINK1 overexpression on ER stress-mediated apoptosis in PD (Fig. 5B). In summary, we conclude that BAP31 is phosphorylated by PINK1 at Ser142, which alleviates PD by inhibiting ER stress-mediated apoptosis.

## Discussion

Emerging evidence suggests that the accumulation of α-synuclein, which is the key pathological hallmark of PD, in the ER may cause ER stress and consequently activate the UPR. The activation of the UPR might in turn be implicated in the regulation of cell death and survival of dopaminergic neurons [17, 33–36]. However, the exact mechanisms that lead to altered ER stress in PD are not clear. BAP31, as a chaperone protein highly expressed in the ER, plays a crucial role in regulating ER stress and apoptosis [22].

Full-length BAP31 can suppress apoptosis through interactions with Bcl-2 or B-cell lymphoma extra-large (Bcl-XL) [37]. BAP31 deficiency induces apoptosis and ER stress by increasing the levels of ER stress-related proteins (e.g., GRP78 and CHOP) and the cytoplasmic calcium concentration in colorectal cancer (CRC) cells [38]. Moreover, BAP31 is highly abundant in neurons and has been implicated in neurodegenerative diseases. Mutations in BAP31 are associated with a severe X-linked phenotype that is characterized by central hypomyelination, dystonia, and deafness [29], whereas BAP31 deficiency is related to amyloid-β plaque formation in AD [28]. On the basis of these findings, we are interested in whether BAP31 is involved in PD pathogenesis. As expected, BAP31 expression was dramatically decreased in PD, and immunofluorescence staining confirmed this finding. Furthermore, overexpression of BAP31 dramatically improved motor dysfunction, memory impairment and TH-positive cell loss in PD mice, and notably increased the fluorescence of BAP31 in vitro. Conclusively, our findings firstly demonstrate the neuroprotective effect of BAP31 in PD.

In order to further validate the mechanism of BAP31 in PD, we evaluated critical indicators of ER stress and apoptosis. Our results revealed that GRP78, CHOP, and Bax expression were notably elevated, whereas Bcl-2 expression was markedly suppressed in the PD model. The overexpression of BAP31 significantly alleviated PD injury by decreasing the expression of ER stress and pro-apoptotic markers (GRP78, CHOP, and Bax) and increasing anti-apoptotic gene Bcl-2 expression. Conversely, knockdown of BAP31 reversed the neuroprotective effects described above. These results suggest that the overexpression of BAP31 suppresses ER stress-mediated apoptosis, thereby attenuating PD injury. Furthermore, 4-PBA (an ER stress inhibitor) significantly inhibited ER stress-induced apoptosis caused by BAP31 knockdown in PD. Notably, the neuroprotective effect of BAP31 was significantly enhanced, as evidenced by decreased GRP78, CHOP, and Bax expression and increased Bcl-2 expression. Consistently, TUNEL assay results demonstrated that 4-PBA alleviated the detrimental effects of BAP31 knockdown on PD.

Considering the pivotal role of BAP31 in the pathogenesis of PD by regulating ER stress-induced apoptosis, how to regulate BAP31 could be of great significance for the prevention and treatment of PD. PINK1 is one of the pathogenic genes associated with autosomal recessive early-onset Parkinson’s syndrome. As a mitochondrial serine/threonine protein kinase, PINK1 can phosphorylate specific substrate proteins to regulate multiple mitochondrial functions, including mitochondrial dynamics, stress response, apoptosis, and adenosine triphosphate (ATP) production [39, 40]. A previous study revealed that PINK1 is involved in the pathogenesis of PD by affecting neuronal survival through the phosphorylation of neuronal substrate proteins [31]. Indeed, PINK1-mediated Drp1(S616) phosphorylation regulates mitochondrial fission, thereby modulating synaptic circuit maturation and synaptic plasticity for memory formation and consolidation [32]. Importantly, PINK1 phosphorylates ER stress-regulating molecules, facilitating their translocation to the nucleus and enhancing the transcriptional activity of ER stress molecules, which further promotes the transcription of PINK1 [41] and the phosphorylation of anti-apoptotic proteins that prevent neuronal cell death, exerting neuroprotective effects [42]. Given this context, it is reasonable to hypothesize that PINK1 could modulate BAP31 to regulate ER stress-induced apoptosis. Surprisingly, BAP31 contains several phosphorylated amino acid sites, and bioinformatics analysis shows that BAP31 is a new substrate protein of PINK1. Therefore, we hypothesize that PINK1 phosphorylates BAP31 to regulate ER stress-induced apoptosis in PD.

As predicted, the interaction of PINK1 with BAP31 was confirmed by Co-IP. Simultaneously, PINK1 overexpression significantly increased the level of BAP31 phosphorylation in vitro. In addition, to identify the sites of BAP31 phosphorylation, we mutated the serine 142 and 216 residues of BAP31. The results showed that overexpressed PINK1 did not affect BAP31 expression when its S142A is mutated, indicating that Ser142 is a crucial amino acid site for PINK1-induced BAP31 phosphorylation. Then, we co-transfected PINK1-overexpressing and BAP31 knockdown plasmids in vitro. As anticipated, the protective effects of PINK1 overexpression on ER stress-mediated apoptosis were abolished by BAP31 interference. Furthermore, we transfected the PINK1-overexpressing plasmid with the BAP31-WT, BAP31-S142A, or BAP31-S142E plasmids into PC12 cells in PD. The results revealed that BAP31-WT and BAP31-S142E protected against ER stress-mediated apoptosis via PINK1 overexpression, whereas BAP31-S142A abolished these effects in PD. On the basis of these data, we conclude that PINK1 phosphorylates BAP31 at the Ser142 residue, and that the interaction between PINK1 and BAP31 is indispensable for regulating ER stress-mediated apoptosis in PD.

There were still several limitations of this study. First, while we demonstrated the neuroprotective role of the PINK1/BAP31 pathway in suppressing ER stress-induced apoptosis, these findings were derived solely from PD mouse models and PC12 cells. Validation of our findings in human induced pluripotent stem cell (iPSC)-derived midbrain dopaminergic neurons from PD patients would be critical for clinical extrapolation. Second, how BAP31 exerts neuroprotective effects through ER-mitochondria crosstalk in PD is unclear. Systematic investigations using techniques such as super-resolution live-cell imaging may track real-time organelle interactions. Finally, exploring the interplay between BAP31 and other PD-associated proteins (e.g., α-synuclein and DJ-1) may contribute to the study of PD pathogenesis.

**Fig. 6:**
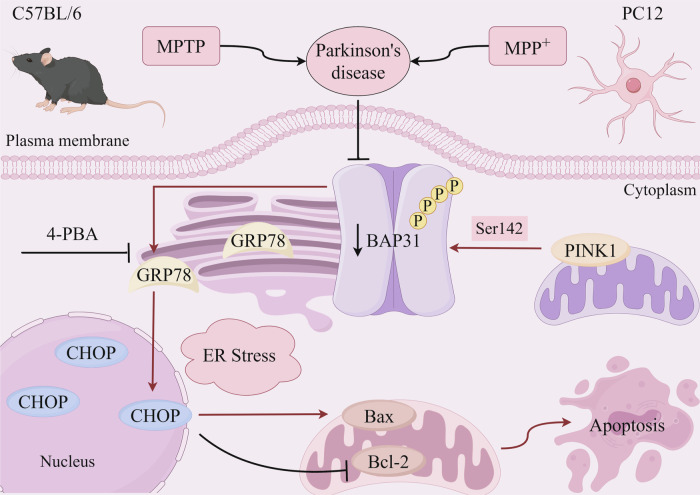
Schematic diagram of the PINK1/BAP31 axis regulating ER stress-induced apoptosis affecting PD. The schematic shows that PINK1 phosphorylates BAP31 at Ser 142 and inhibits ER stress-induced apoptosis by regulating BAP31 to alleviate PD. Created with Figdraw.

In summary, our current study demonstrated that BAP31 significantly suppresses ER stress-mediated apoptosis and alleviates PD. Furthermore, PINK1 interacts with BAP31 and phosphorylates BAP31 at the Ser142 residue. Mechanistically, the protective effects of PINK1 overexpression on attenuating ER stress-induced apoptosis are abolished by BAP31 knockdown, indicating that the interaction between PINK1 and BAP31 is indispensable for regulating ER stress-mediated apoptosis in PD (Fig. 6). Our study provides novel insight into mitigating dopaminergic neuronal loss in PD pathogenesis by regulating the PINK1/BAP31 signaling axis.

## Materials and methods

### Animal experiments

#### Mouse model of PD

Male C57BL/6 mice (8–12 weeks of age) weighing 22–25 g were provided with standard laboratory food nutrition and water. The mice were randomly divided into groups (10 mice/group), and the investigators were blinded to the experiment performance. The PD model was constructed by intraperitoneal injection of 30 mg/kg MPTP (Sigma Aldrich, Missouri, USA) for 5 consecutive days. The control group was administered the corresponding volume of saline. After the experiment, the mice were executed and the brain tissues were collected for testing.

#### Brain stereotaxic injection

Male C57BL/6 mice were anesthetized by intraperitoneal injection of 2.5% 2, 2, 2-tribromoethanol (Macklin, Shanghai, China) and fixed on a brain stereotactic apparatus (Zongshi Dicheng, Beijing, China). A stereotaxic injection of 2 μl of adeno-associated virus (AAV) (AAV2/9-control or AAV2/9-BAP31) was injected into the bilateral SN region (AP: −3 mm; ML: ±1.25 mm; DV: −4.5 mm). The injection rate was 1 µL/min. The needle remained in place for 8 min before it was withdrawn slowly. The viral preparations had the following titres: AAV2/9-control (1.8 × 10^12^ vg/ml), AAV2/9-BAP31 (1.2 × 10^12^ vg/ml) (Hanbio, Shanghai, China). Four weeks later, PD model mice were treated with MPTP as described above. Subsequently, animal behavioral tests were performed. Mice exhibiting abnormal behavior during acclimation were excluded. All the procedures were conducted in compliance with the Guidelines for the Management and Use of Laboratory Animals for animal experiments, and were approved by the Institutional Ethics Committee of Dalian Medical University.

### Behavioral tests

#### Rotarod test

The rotarod test is used to assess both motor coordination ability and balance ability in mice. The rotarod has 6 channels, and the rotor diameter is 30 mm. The mice were acclimated for 3 days before the test. The rotation time of the rotor was 300 s. The rotational speed was set at 10 rpm/min for the first 1 min and then increased from 10 rpm/min to 40 rpm/min for the last 4 min. Each mouse was tested 3 times. The interval between each test was more than 30 min. The fall-time data were recorded. The mice were placed in the experimental environment for more than 30 min in advance. (Zhenghua Bio Instrumentation, Anhui, China).

#### Novel object recognition experiment

This test evaluated the memory capacity of the mice and was divided into three parts. First, there was an acclimatization period, during which each mouse was free to explore the experimental setup without gadgets for 5 min. Second, the familiarization phase, in which two gadgets of identical shape and size were put into the setup, and each mouse was familiarized with them for 5 min. Finally, there was a formal testing phase, in which one of the objects was replaced with a different object in the device (AC or BC). Similarly, each mouse was allowed to explore for 5 min. The time spent exploring the old and novel objects was recorded. Discrimination index = (time spent exploring novel objects/total exploration time) × 100% (Gizmos Software Technology, Shanghai, China).

### Cell culture and transfection

PC12 cells were cultivated in DMEM medium (Solabao, Beijing, China) containing 10% fetal bovine serum (FBS), 100 μg/mL penicillin, and 100 μg/mL streptomycin in a humidified environment of 5% CO_2_ and 95% O_2_ at 37 °C (Thermo, Waltham, Massachusetts, USA). The si-BAP31, BAP31-WT, BAP31-S142A, and BAP31-S142E plasmids were obtained from GenePharma (Suzhou, China). PC12 cells were transfected with plasmids or siRNA using Lipofectamine 3000 (Invitrogen, California, USA) for 24 h and then treated with 4-PBA (10 μM) for 4 h (Macklin, Shanghai, China) and MPP^+^ (1.5 mM) for 24 h (Macklin, Shanghai, China). The interference sequences of BAP31 were as follows: si-BAP31, sense (5’ to 3’)-GGUUCUCAUCGUCAUCCUUTT and antisense (5’ to 3’)-AAGGAUGACGAUGAGAACCTT.

### Immunofluorescence analysis

TH immunofluorescence was performed according to protocols described in previous research [43]. Briefly, frozen sections of SN samples (20 μm) were incubated with 10% regular goat serum containing 0.3% Triton X-100 for 1.5 h at 37 °C. Subsequently, the sections were incubated with an anti-TH antibody (1:250, ABclonal, Wuhan, China) for 24 h at 4 °C and then with goat anti-rabbit polyclonal antibody (1:200, Proteintech, Wuhan, China) for 1.5 h at 37 °C. Images were acquired with a fluorescence microscope (Olympus, Tokyo, Japan). The numbers of TH-positive cells were quantified using ImageJ software.

The cells were stabilized and permeabilized with 4% paraformaldehyde (Biosharp, Beijing, China) supplemented with 0.2% Triton X-100 for 20 min. The cells were incubated with using 5% goat serum sealing solution (Solabao, Beijing, China) for 1 h. A diluted anti-BAP31 antibody (1:100, Proteintech, Wuhan, China) was applied dropwise to the cells, which were subsequently incubated overnight at 4 °C in the dark. A fluorescent secondary antibody (1:200, Proteintech, Wuhan, China) was applied, and the samples were incubated for 1 h in the dark. An appropriate amount of anti-fluorescence quencher (containing DAPI) (Severn Innovation, Beijing, China) was added to each slide (Zhongsui Jinqiao, Beijing, China). A fluorescence microscope was used to visualize the slides (Carl Zeiss, Jena, Germany).

### Co-immunoprecipitation

An antibody was incubated with Protein A/G immunoprecipitation beads (Selleck, Houston, Texas, USA) overnight at 4 °C, followed by incubation of the protein supernatant with Protein A/G immunoprecipitation beads at room temperature for 3 h. After incubation, the supernatant was discarded, and the protein was denatured. The beads were then discarded, and the supernatant was stored.

### Western blotting

Protein samples from mouse SN and PC12 cells were lysed in 1% PMSF and IP lysis buffer (Beyotime, Shanghai, China). The supernatants were collected, and the protein concentrations were analyzed with a BCA kit (Beyotime, Shanghai, China). The proteins were separated using 10–15% SDS-PAGE and then transferred to PVDF membranes (Millipore, Boston, Massachusetts, USA). After being blocked with 5% skimmed milk powder, the PVDF membranes were incubated overnight at 4 °C with the following antibodies: anti-BAP31 (11200-1-AP), anti-PINK1 (23274-1-AP), anti-Bax (60267-1-Ig), anti-Bcl-2 (68103-1-Ig), anti-GRP78 (66574-1-Ig), anti-CHOP (66741-1-Ig) and anti-IgG (30000-0-AP) from Proteintech (Wuhan, China); anti-PINK1 (507131) and anti-p-Tyrosine (R381703) from Zhengneng (Chengdu, China); anti-p-Serine/Threonine (AP0893) from ABclonal (Wuhan, China); and anti-β-actin (F0012) from Selleck (Houston, Texas, USA). The secondary antibody reaction was performed with goat anti-mouse (SA00001-1) or goat anti-rabbit (SA00001-2) secondary antibodies from Proteintech (Wuhan, China) at a dilution of 1:2000 for 1.5 h at 37 °C. Images were acquired using an Amersham Imager 600 visualizer. Optical density analysis was performed using ImageJ software.

### TUNEL assay

Apoptosis induced by MPP^+^ and 4-PBA was detected with the One-step TUNEL In Situ Apoptosis Kit (Abbkine, Wuhan, China). PC12 cells were fixed and permeabilized with 4% paraformaldehyde and Triton X-100. Then, the adherent cells were stained with the TUNEL reaction mixture and an anti-fluorescence quencher containing DAPI. The staining was visualized by a fluorescence microscope (Carl Zeiss, Jena, Germany).

### Statistical analysis

All the data were analyzed as mean ± standard deviation (SD) by GraphPad Prism 9.5 software. Student’s unpaired t-test was used to compare the means between two groups, and one-way analysis of variance (ANOVA) was used to analyze the means among several groups, followed by a Tukey’s post-hoc test. A *P* value  <  0.05 was considered statistically different.

## Supplementary information

Original Western blots

Checklist

## Data Availability

The datasets used and/or analyzed during the current study are available from the corresponding author on reasonable request.
