# Supplementary material for: BAP31 represses endoplasmic reticulum stress-mediated apoptosis and alleviates neurodegeneration in Parkinson’s disease
Source: Cell Death Dis. 2025 Sep 29;16(1):672. doi: 10.1038/s41419-025-07907-3 (PMC12480763; doi:10.1038/s41419-025-07907-3)
Supplement: Supplementary file 1 — Original Western blots [file 41419_2025_7907_MOESM1_ESM.pdf]

Figure 1A	Control MPTP
BAP31	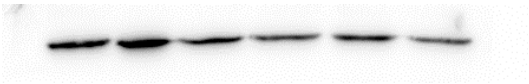
β-actin	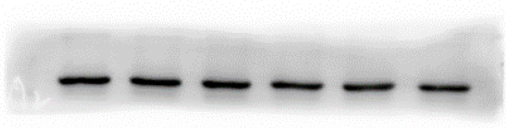
Figure 1C	Control MPP+
BAP31	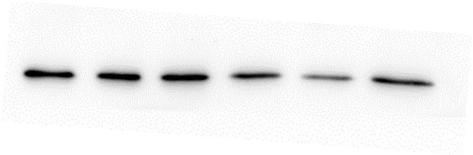
β-actin	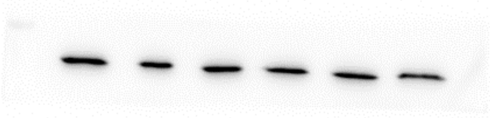
Figure 3A	Control MPTP
GRP78	100KDa → 70KDa → 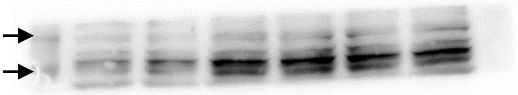
CHOP	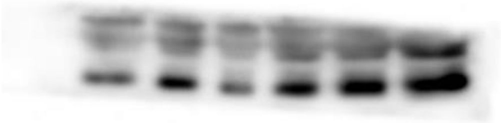
Bcl-2	35KDa → 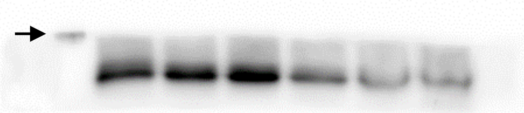
Bax	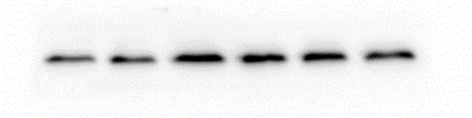
β-actin	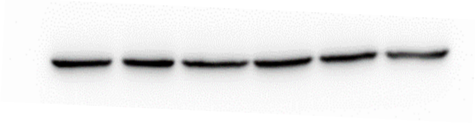
Figure 3B	Control MPP+

GRP78	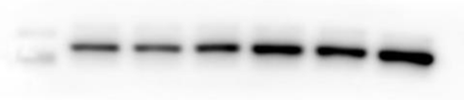
CHOP	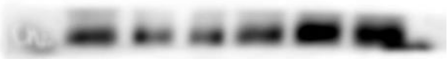
Bcl-2	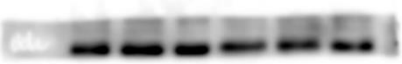
Bax	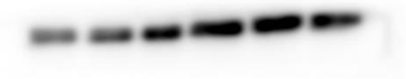
β-actin	55KDa → 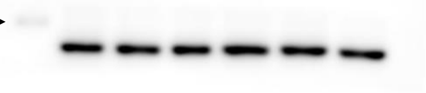
Figure 3C	[Control] [Control+AAV2/9-BAP31] [MPTP] [MPTP+AAV2/9-BAP31]
BAP31	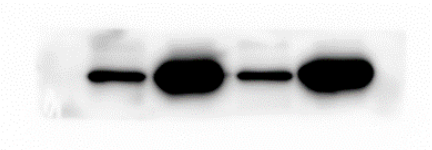
GRP78	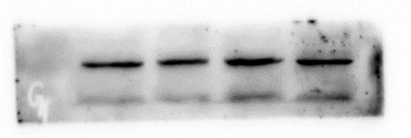
CHOP	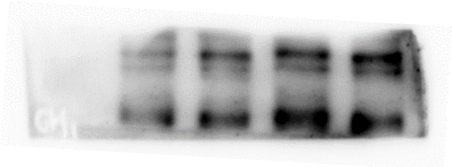
Bcl-2	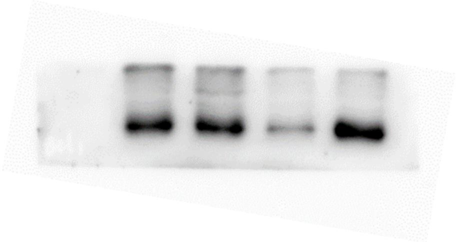

Bax	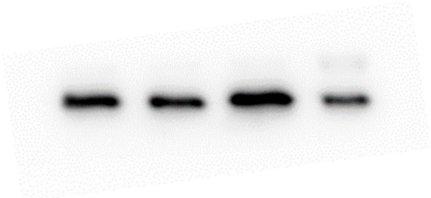
β-actin	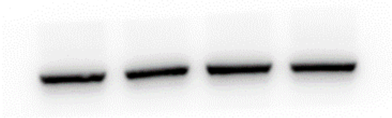
Figure 3D	[pcDNA3.1] [pc-BAP31]<br>[MPP^++pcDNA3.1] [MPP^++pc-BAP31]
BAP31	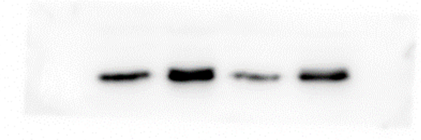
GRP78	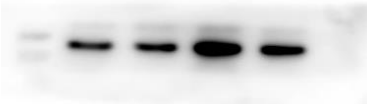
CHOP	35KDa → 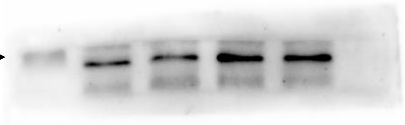
Bcl-2	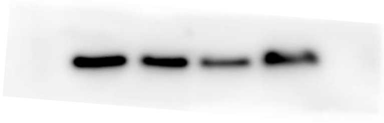
Bax	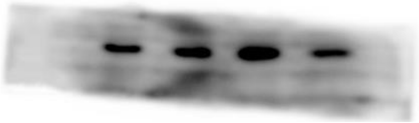
β-actin	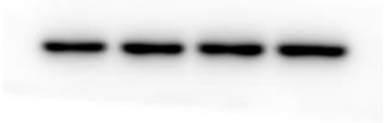
Figure 3E	[si-control] [si-BAP31]<br>[MPP^++si-control] [MPP^++si-BAP31]
BAP31	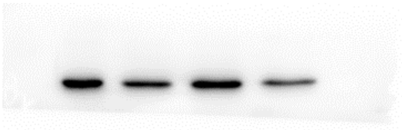

GRP78	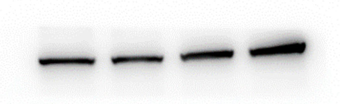
CHOP	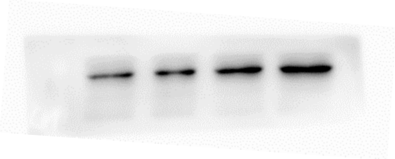
Bcl-2	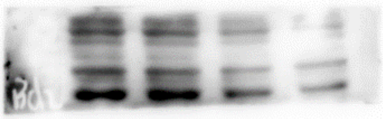
Bax	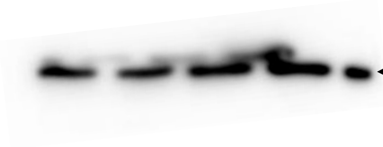 ← 20KDa
β-actin	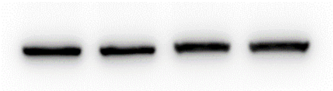
Figure 3F	[si-control] [MPP^++si-control]<br>[MPP^++si-BAP31] [MPP^++si-BAP31+4-PBA]
GRP78	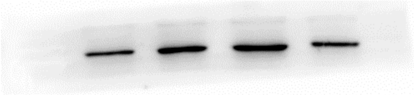
CHOP	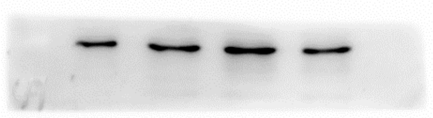
Bcl-2	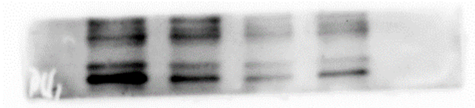
Bax	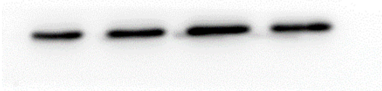
β-actin	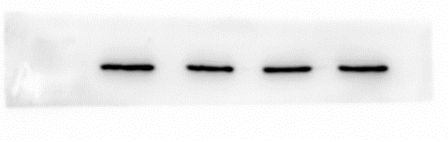
Figure 4A	Control MPTP

PINK1	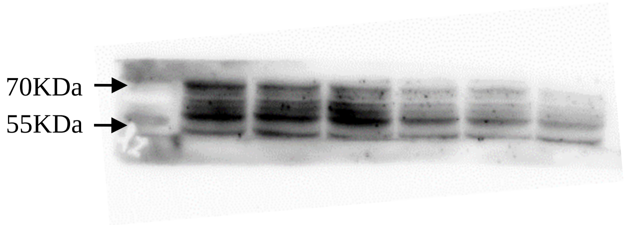
β-actin	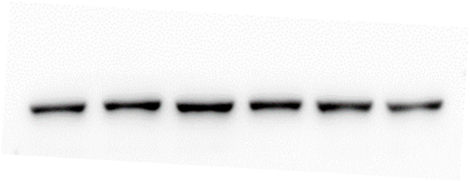
Figure 4B	Control MPP$^+$
PINK1	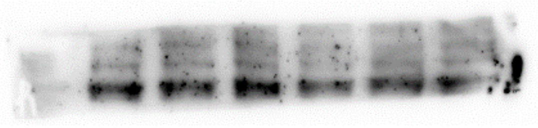
β-actin	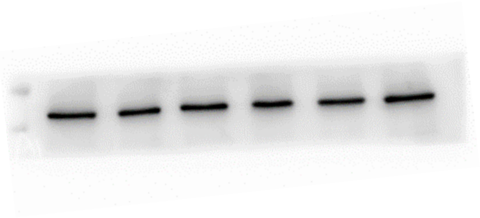
Figure 4G	Input IgG PINK1(Co-IP)
BAP31	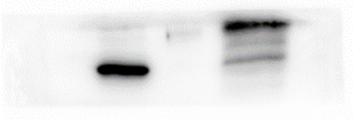
PINK1	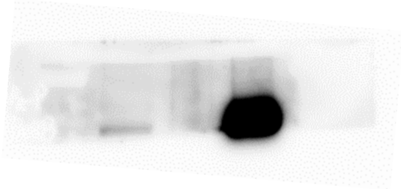
Figure 4H	Input IgG BAP31(Co-IP)
BAP31	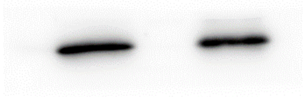
PINK1	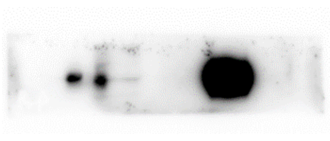
Figure 4I	Control MPP$^+$

p-Ser/Thr	70KDa → 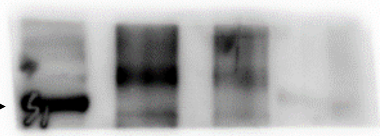
p-Tyr	70KDa → 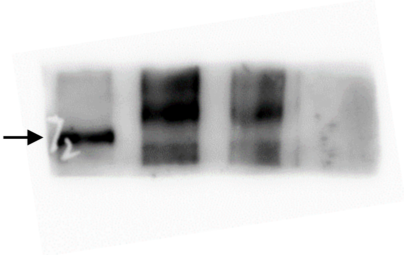
BAP31	35KDa → 25KDa → 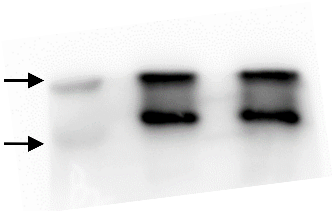
Figure 4J	Control pc-PINK1(IP)
p-Ser/Thr	100KDa → 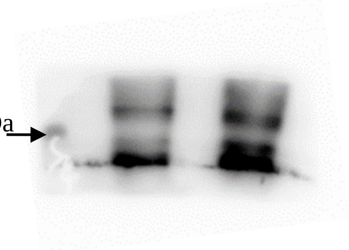
BAP31	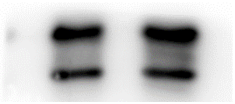
Figure 4J	Control pc-PINK1(Input)
PINK1	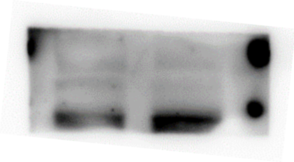
BAP31	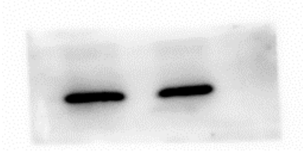
β-actin	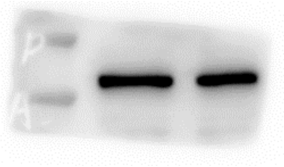

Figure 5M	[BAP31 WT] [pc-PINK1+BAP31 WT]<br>[BAP31 S142A] [pc-PINK1+BAP31 S142A]<br>[BAP31 S216A] [pc-PINK1+BAP31 S216A] (IP)
p-Ser/Thr	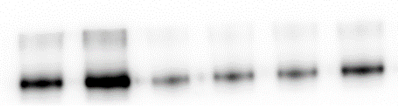
BAP31	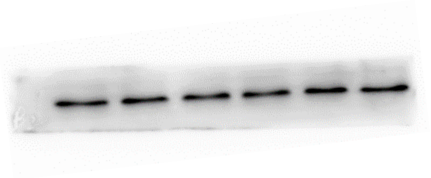
Figure 5M	[BAP31 WT] [pc-PINK1+BAP31 WT]<br>[BAP31 S142A] [pc-PINK1+BAP31 S142A]<br>[BAP31 S216A] [pc-PINK1+BAP31 S216A] (Input)
PINK1	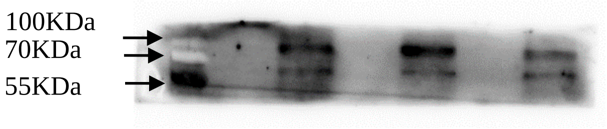
β-actin	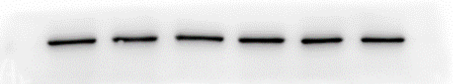
Figure 5A	[pcDNA3.1]<br>[MPP$^+$+pcDNA3.1]<br>[MPP$^+$+pc-PINK1]<br>[MPP$^+$+pc-PINK1+si-control]<br>[MPP$^+$+pc-PINK1+si-BAP31]
PINK1	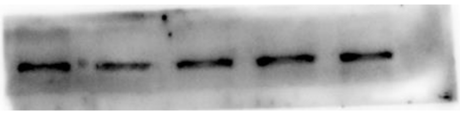
BAP31	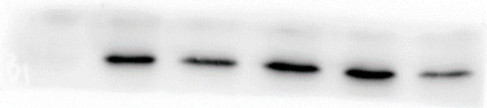
GRP78	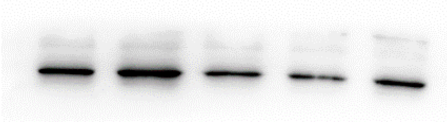
CHOP	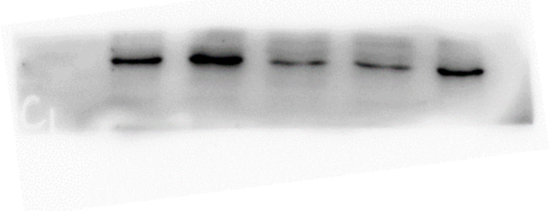

Bcl-2	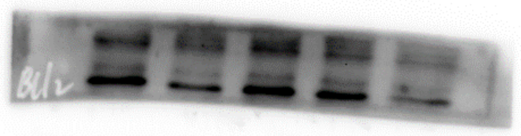
Bax	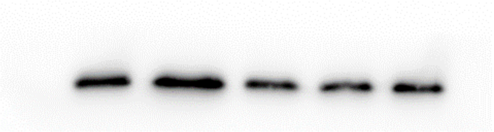
β-actin	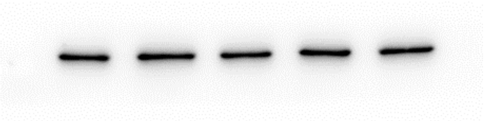
Figure 5B	[pcDNA3.1]<br>[MPP^{+}+pcDNA3.1]<br>[MPP^{+}+BAP31 WT]<br>[MPP^{+}+BAP31 WT+pc-PINK1]<br>[MPP^{+}+pc-PINK1+BAP31 S142A]<br>[MPP^{+}+pc-PINK1+BAP31 S142E]
PINK1	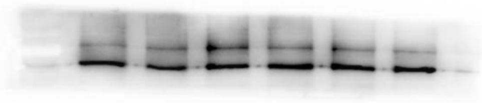
BAP31	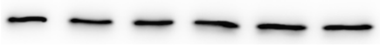
GRP78	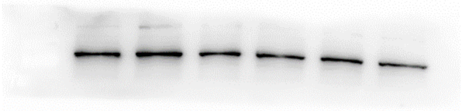
CHOP	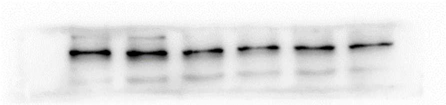
Bcl-2	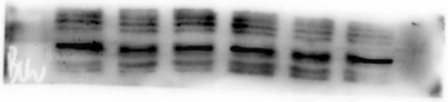
Bax	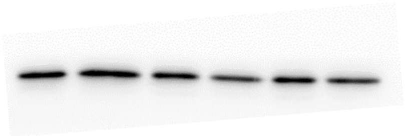
β-actin	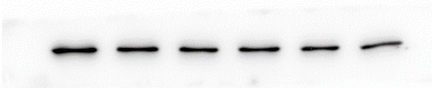
